# Neuropathogenicity of non-viable *Borrelia burgdorferi *ex vivo

**DOI:** 10.1038/s41598-021-03837-0

**Published:** 2022-01-13

**Authors:** Geetha Parthasarathy, Shiva Kumar Goud Gadila

**Affiliations:** 1 Division of Immunology, Tulane National Primate Research Center, Tulane University, 18703, Three Rivers Road, Covington, LA 70433 USA; 2 Tulane National Primate Research Center, 18703, Three rivers Road, Room 109, Covington, LA 70433 USA

**Keywords:** Cell biology, Immunology, Microbiology, Neuroscience

## Abstract

Even after appropriate treatment, a proportion of Lyme disease patients suffer from a constellation of symptoms, collectively called Post-Treatment Lyme Disease Syndrome (PTLDS). Brain PET scan of patients with PTLDS have demonstrated likely glial activation indicating persistent neuroinflammatory processes. It is possible that unresolved bacterial remnants can continue to cause neuroinflammation. In previous studies, we have shown that non-viable *Borrelia burgdorferi* can induce neuroinflammation and apoptosis in an oligodendrocyte cell line. In this follow-up study, we analyze the effect of sonicated remnants of *B. burgdorferi* on primary rhesus frontal cortex (FC) and dorsal root ganglion (DRG) explants. Five FC and three DRG tissue fragments from rhesus macaques were exposed to sonicated *B. burgdorferi* and analyzed for 26 inflammatory mediators. Live bacteria and medium alone served as positive and negative control, respectively. Tissues were also analyzed for cell types mediating inflammation and overall apoptotic changes. Non-viable *B. burgdorferi* induced significant levels of several inflammatory mediators in both FC and DRG, similar to live bacteria. However, the levels induced by non-viable *B. burgdorferi* was often (several fold) higher than those induced by live ones, especially for IL-6, CXCL8 and CCL2. This effect was also more profound in the FC than in the DRG. Although the levels often differed, both live and dead fragments induced the same mediators, with significant overlap between FC and DRG. In the FC, immunohistochemical staining for several inflammatory mediators showed the presence of multiple mediators in astrocytes, followed by microglia and oligodendrocytes, in response to bacterial remnants. Staining was also seen in endothelial cells. In the DRG, chemokine/cytokine staining was predominantly seen in S100 positive (glial) cells. *B. burgdorferi* remnants also induced significant levels of apoptosis in both the FC and DRG. Apoptosis was confined to S100 + cells in the DRG while distinct neuronal apoptosis was also detected in most FC tissues in response to sonicated bacteria. Non-viable *B. burgdorferi* can continue to be neuropathogenic to both CNS and PNS tissues with effects likely more profound in the former. Persistence of remnant-induced neuroinflammatory processes can lead to long term health consequences.

## Introduction

Lyme disease (LD), caused by the bacterium *Borrelia burgdorferi* is the leading vector borne illness in the US and northern hemisphere. According to the latest estimates, the incidence of LD is approximately 476,000 cases per year^1^. Bacterial transmission occurs through tick bite, followed typically by appearance of a bulls-eye type rash called erythema migrans that appears at the site of tick attachment. This early localized phase is generally accompanied by fever, headache, and malaise, followed by an early dissemination phase affecting multiorgan systems. These include other skin areas, heart, joints and the nervous system, both central and peripheral. Early Lyme disease is treated successfully with antibiotics in most cases, when diagnosed early and treated appropriately^2^. However, about 10–35% of patients treated for erythema migrans or early LD have persistent or intermittent musculoskeletal, cognitive, or fatigue complaints of mild to moderate intensity at 6 to 12 months of follow up^3–5^. Other notable symptoms include, joint pain, headache, lower back pain, irritability, paresthesia, sleep issues and depression^3^. These symptoms are collectively termed Post-Treatment Lyme Disease Syndrome or PTLDS. In a recent study, positron emission tomography (PET) scan of PTLDS patients with radiotracer [C^11^]DPA-713 showed likely glial activation in the brain many months after treatment^6^. This radiotracer can bind/quantify cerebral TSPO, an 18-KDa translocator protein that shows increased expression in reactive astrocytes and activated microglia. The results of the study indicated that unresolved neuroinflammatory processes can persist in a cohort of patients with PTLDS.

The underlying pathophysiological mechanisms governing the PTLDS symptoms are the subject of intense controversy. Several theories have been put forward including persistent organisms impervious or inaccessible to antibiotics^7,8^, autoimmunity to bacterial antigens^9^, coinfections with other tick-borne pathogens^10^, post-infective fatigue syndromes, and microbiome changes^11^. In addition to these, in our previous study we showed that non-viable *B. burgdorferi*, (a state that can be achieved naturally in vivo or through the action of antibiotics), can cause inflammation and apoptosis in an oligodendrocyte cell line MO3.13^12^. Primary human microglia have also been shown to induce chemokines and cytokines in response to antibiotic killed or sonicated *B. burgdorferi*^13^. This suggested that in the subset of patients with persistent glial activation, residual antigens from antibiotic treatment, could be a source of this neuroinflammation.

Since our and other in vitro studies used immortalized oligodendrocytes or primary microglia alone, it was not clear if such a phenotype can occur in primary brain tissues in response to non-viable *B. burgdorferi*. It is possible, for instance, that this neuroinflammatory effect is masked in a tissue setting in the presence of other cells. Also, as DRG plays a role in neuropathic pain^14^, in this follow-up study, we analyzed the neuropathological effect of sonicated *B. burgdorferi* not only on primary rhesus frontal cortex tissues but also primary rhesus dorsal root ganglion tissues, sourced from multiple animals. Live *B. burgdorferi* was used as a positive control for induction of neuroinflammation and apoptosis. The results show that like our in vitro study on the oligodendrocyte cell line, non-viable *B. burgdorferi* is neuropathological to both central and peripheral nervous system (CNS and PNS) primary tissue explants, with effects beyond the oligodendrocytes.

## Methods

### Bacterial strain and culture

*B. burgdorferi* strain B31, clone 5A19, was used throughout the course of this study. This strain possesses the full complement of plasmids required for infectivity and pathogenicity and was routinely cultured in Barbour-Stoenner-Kelly (BSK-H) medium (Sigma Aldrich, St. Louis-MO) supplemented with amphotericin (0.25 µg/mL), phosphomycin (193 µg/mL) and rifampicin (45.4 µg/mL), for about 5–6 days, under microaerophilic conditions. Concentration of bacteria was determined using a dark field microscope, and the required number was harvested by centrifugation at 2095×*g* for 30 min at room temperature, without brakes.

Bacteria was resuspended to the original concentration in RPMI 1640 medium (BioWhittaker, Fisher Scientific, Waltham, MA) supplemented with 10% fetal bovine serum (FBS) (Hyclone, GE Lifesciences, Pittsburgh, PA) and subsequently diluted in the same medium to the desired concentrations. Non-viable bacteria were obtained by sonication according to previously published protocols^12^. Briefly, after resuspending the bacterial pellet in RPMI 1640, volume equivalent of live bacteria was sonicated at 40% pulse, 15% amplitude 15 s each, 7 times with a sonicator (Model 120, Fisher Scientific) and diluted in RPMI 1640 to the required equivalent concentration. Non-viability of the sonicated bacteria was verified by microscopy before each experiment.

### Ex vivo assays

Frontal cortex (FC) and dorsal root ganglion (DRG) tissues were obtained from uninfected rhesus macaques (*Macaca mulatta*) that were euthanized from the breeding colony due to persistent diarrhea, or injury. Euthanasia was performed according to the protocols recommended and approved by the Tulane Institutional Animal Care and Use Committee. Assays were performed according to previously published protocols^15^. Briefly, freshly obtained FC tissues were sliced in a tissue slicer (Ted Pella Inc., Redding, CA) to approximately 2 mm thickness each and transferred to 12 well tissue culture plates containing RPMI 1640 with 10% FBS (Experimental medium, EM). The medium was replaced with fresh EM containing 1 × 10^7^/mL of live *B. burgdorferi* or its sonicated equivalent in the presence or absence of Brefeldin A at a final concentration of 10 µg/mL (Molecular Probes, Eugene, OR). The latter is a fungal metabolite that prevents protein transport and enhances visualization of intracellular proteins during immunohistochemistry. As DRG tissues are very small, individual DRG or sections halved with a scalpel were used. The tissues were cocultured with the bacteria/sonicated fragments for 4 h at 37 °C, 5% CO_2_. Tissues in EM alone (with or without Brefeldin A) were used as Medium (negative) controls, while live bacteria were used as a positive control for chemokine/cytokine and apoptosis induction. Supernatants were collected, centrifuged at 2095×*g*, for 10 min at 4 °C, transferred to O-ring collection tubes (Sarstedt Inc., Newton, NC) and stored at − 20 °C, until analysis by Multiplex ELISA. Tissues were fixed in 2% paraformaldehyde overnight, washed and transferred to 30% sucrose for 2–3 days, cryopreserved in O.C.T (optimal cutting temperature) compound and stored at − 80 °C until sectioning.

### Quantitation of chemokines and cytokines

Supernatants from the ex vivo assays were analyzed for IL-6, CXCL8, CCL2 by Enzyme-Linked Immuno-Sorbent assays (ELISAs) using custom non-human primate (NHP)-Procartaplex kits from Lifetechnology Corporation (Grand Island, NY), at the Pathogen Detection and Quantification Core, Tulane National Primate Research Center, and performed according to the manufacturer’s protocols. An additional custom 23-plex NHP chemokine/cytokine kit (Procartaplex, Lifetech Corp.) was used to analyze other chemokines/cytokines induced in response to live or non-viable *B. burgdorferi* ex vivo. The included analytes were BDNF, BLC (CXCL13), FGF-2, G-CSF (CSF-3), GM-CSF, IFN γ, IL-1β, IL-1RA, IL-4, IL-7, IL-10, IL-12p70, IL-15, IL-17A(CTLA-8), IL-18, IL-23, IP-10 (CXCL10), I-TAC (CXCL11), MIP-1α (CCL3), NGFβ, SCF, TNFα and VEGF-A. IL-6 levels in some samples were quantified using monkey IL-6 calorimetric ELISA kit according to the manufacturer’s instructions (Thermofisher Scientific, Waltham, MA). The results were graphed using Microsoft Excel® and figures were assembled using Microsoft Powerpoint® and Adobe® Photoshop CS6.

### Terminal deoxynucleotidyl transferase dUTP nick-end labeling (TUNEL) assay

The effect of non-viable *B. burgdorferi* on cell viability of tissues was determined by TUNEL assay. Cryosections, approximately 7–10 µm thick, were stained for neuron specific MAP2, and the TUNEL assay was performed thereafter following the manufacturer’s instructions (EMD Millipore Apoptag Fluorescein kit), and as described in Ramesh et al.^16^. An anti-human mouse monoclonal against human MAP2 (1:50) was used as primary antibody. Appropriate secondary antibody conjugated to Alexa Fluor 568 (1:1000; Thermofisher Scientific, Waltham, MA) was used to visualize neurons. At the end of the assay, the slides were mounted with an anti-quenching reagent, cover-slipped and visualized under a fluorescent microscope. To obtain quantitative data, for each section apoptotic nuclei were counted in 10–20 fields. Average apoptotic nuclei per field was calculated for each treatment and graphed using Microsoft Excel® software.

### Immunohistochemistry

Cryosections (7–10 µm thick) were permeabilized in phosphate buffered saline (PBS) containing 0.1% Triton-X-100, blocked with 10% normal goat serum (NGS) for 1 h, followed by staining for IL-6, IL-8 (CXCL8) or MCP-1(CCL2) along with microglia (Iba1), oligodendrocyte (MBP), astrocyte (GFAP) or neuron (MAP2 or NeuN) specific cell markers. The following primary antibodies were used: anti-human rat [(1:20), BD biosciences, Franklin Lakes, NJ] or mouse IL-6 [(1:1000) ProSpec, East Brunswick, NJ], anti-human rabbit (Fitzgerald Industries, Acton, MA) or mouse (Thermofisher Scientific, Waltham, MA) IL-8 (1:20); anti-human rabbit or mouse MCP-1 (1:20, Thermofisher Scientific); anti-human rabbit (Wako, Richmond, VA) or mouse (Santa Cruz biotechnology, Dallas, TX) Iba1 (1:50); anti human mouse MAP2 (1:50, Sigma Aldrich, St. Louis, MO); anti-human mouse NeuN (1:10, Millipore Sigma, Burlington, MA); anti-human mouse GFAP-cy3 (1:200, Sigma Aldrich) or anti-human rabbit GFAP (1:50, BioGenex, Fremont, CA); anti- human rabbit MBP (1:1000, Millipore Sigma). Following staining with primary antibody, sections were stained with appropriate secondary species-specific whole IgG antibody conjugated to Alexa Fluor 488 or Alexa Fluor 568 (both 1:1000, Thermofisher Scientific), in PBS containing 10% NGS, for 45 min–1 h each. The nuclei were stained with TOPRO-3 iodide (1:1000; Thermofisher Scientific), or DAPI (1:5000, Millipore Sigma) when required. Tissues were mounted with anti-quenching reagent, covered with coverslip and visualized for cell specific cytokine/chemokine staining.

### Microscopy

Fluorescent microscopy pictures were captured using a Leica DMRE fluorescent microscope (Leica microsystems, Buffalo Grove-IL) and Lumecor SOLA GUI software (Lumencor, Beaverton-OR). Cells were imaged using Nuance Multispectral Imaging System (CRi, PerkinElmer, Waltham-MA). A Nikon Ti2- E fluorescent microscope (Nikon, Melville, NY), with NIS-Elements software (Nikon Instruments) was also used as required. Confocal microscopy was carried out with Leica TCS SP2 confocal microscope (Leica microsystems, Buffalo Grove, IL). Adobe® Photoshop CS6 software was used to assemble the images.

### Statistics

The Student’s t-test (two-tailed) was used to determine the statistical significance of an experimental outcome, with each analysis using duplicate values. The results were considered significantly different if the probability values (p) were ≤ 0.05.

## Results

### Non-viable *B. burgdorferi* induces inflammatory mediators in primary rhesus frontal cortex tissues

**Figure. 1:**
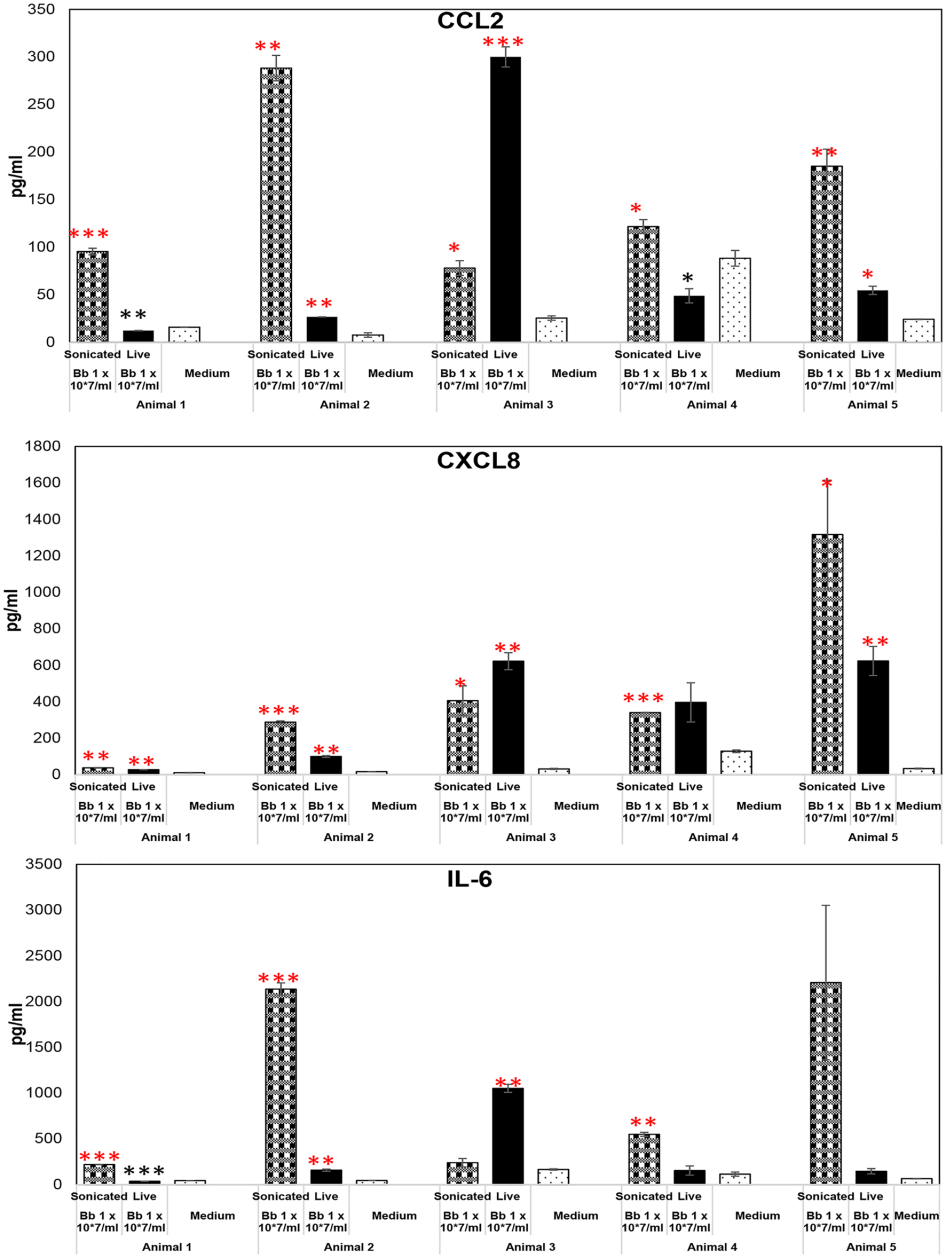
Non-viable *B. burgdorferi* mediates inflammatory mediator release from rhesus FC. The effect of live or sonicated *B. burgdorferi* on inflammatory mediator release from rhesus frontal cortex tissues is shown in this figure. Ex vivo cultured tissues were exposed to indicated treatments for 4 h. Supernatants were analyzed for CCL2, CXCL8 and IL-6. Results from tissues obtained from 5 different animals is shown. ***p < 0.001; **p < 0.01; *p < 0.05. Significantly increased levels over medium control is denoted by the red asterisks, while black asterisks indicate they were below medium levels. Bars represent standard deviation. Live bacteria were used as a positive control, while medium exposed tissues were used as a negative control throughout the study.

Frontal cortex tissues from five different rhesus macaques were used for this study. The animal tissues were sourced from both sexes, varied in age from a little over a year to approximately 16 years of age and had both Indian and Chinese strains (Table 1). Freshly obtained tissues were exposed to live or sonicated *B. burgdorferi* for 4 h, and supernatants were analyzed for CCL2 (MCP-1), CXCL8 (IL-8) or IL-6. As seen in Fig. 1, non-viable *B. burgdorferi* was able to induce higher levels of CCL2, CXCL8 and IL-6 over medium controls in all 5 tissues, in both sexes, strains and across age groups. While these levels were statistically significant for CCL2 and CXCL8 in all 5 tissues, they were so for 3 out of 5 tissues for IL-6. Live bacteria have been shown to induce inflammatory mediators in rhesus tissues previously and hence were used as a positive control^15^. Surprisingly, at the same equivalent concentrations, they were less potent than non-viable *B. burgdorferi* and showed more variations in inflammatory mediator induction. Live bacteria induced significantly higher levels of CCL2 over medium controls in 3/5 tissues, while levels were significantly lower than medium levels in 2/5 (Fig. 1). They induced higher levels of IL-6 (than medium controls) in 4/5 tissues, (being significantly so in 2 of the 4), while being significantly lower than medium levels in 1/5. There was similarity with non-viable *B. burgdorferi* in induction of CXCL8 as higher levels were seen in all 5 tissues, being significantly so in 4/5. These results indicated that non-viable spirochetes are potent and consistent inducers of inflammatory mediators in primary rhesus frontal cortex tissues, and maybe more so than the live ones. To look at this more concretely, we analyzed for differences in inflammatory mediator induction between live and non-viable *B. burgdorferi* treatments, and the results are shown in Table 2A. CCL2 levels induced by non-viable *B. burgdorferi* were significantly higher than those induced by live bacteria in 4/5 animal frontal cortex tissues. IL-6 levels were again higher in those induced by non-viable *B. burgdorferi* in 4/5 tissues (significantly so in 3 of the 4) and 3/5 for CXCL8 (significantly higher in 2 of the 3). Only one animal (#3) showed higher levels induced by live bacteria and was in the only Chinese rhesus tissue. These results indicate that while there are variations in inflammatory mediator induction in outbred animals, non-viable *B. burgdorferi* is more potent and consistent inductor of neuroinflammatory mediators in brain tissues than live bacteria.

**Table 1:** Animals, strain and age used in this study.

Animal number	Age (years)	Sex
1	3.08	Male, Indian rhesus
2	2.26	Male, Indian rhesus
3	15.62	Female, Chinese rhesus
4	1.50	Male, Indian rhesus
5	10.80	Female, Indian rhesus

**Table 2:** Fold change in cytokine levels induced by non-viable *B. burgdorferi* over live bacteria induced levels.

2A: Frontal cortex tissues			
FC	CCL2	CXCL8	IL-6
Animal 1	7.81*** (± 0.13)	1.37* (± 0.04)	6.28*** (± 0.003)
Animal 2	10.83** (± 0.44)	2.90** (± 0.08)	13.67*** (± 0.59)
Animal 3	0.26** (± 0.02)	0.66 (± 0.18)	0.23** (± 0.05)
Animal 4	2.51* (± 0.23)	0.89 (± 0.25)	3.79** (± 1.11)
Animal 5	3.39** (± 0.06)	2.10 (± 0.21)	15.03 (± 2.79)

2B: DRG tissues			
DRG	CCL2	CXCL8	IL-6
Animal 3	0.88 (± 0.18)	1.09 (± 0.17)	2.73* (± 0.04)
Animal 4	1.15 (± 0.16)	0.40* (± 0.02)	0.52* (± 0.04)
Animal 5	1.30** (± 0.02)	1.58** (± 0.03)	1.33* (± 0.02)

**Figure 2:**
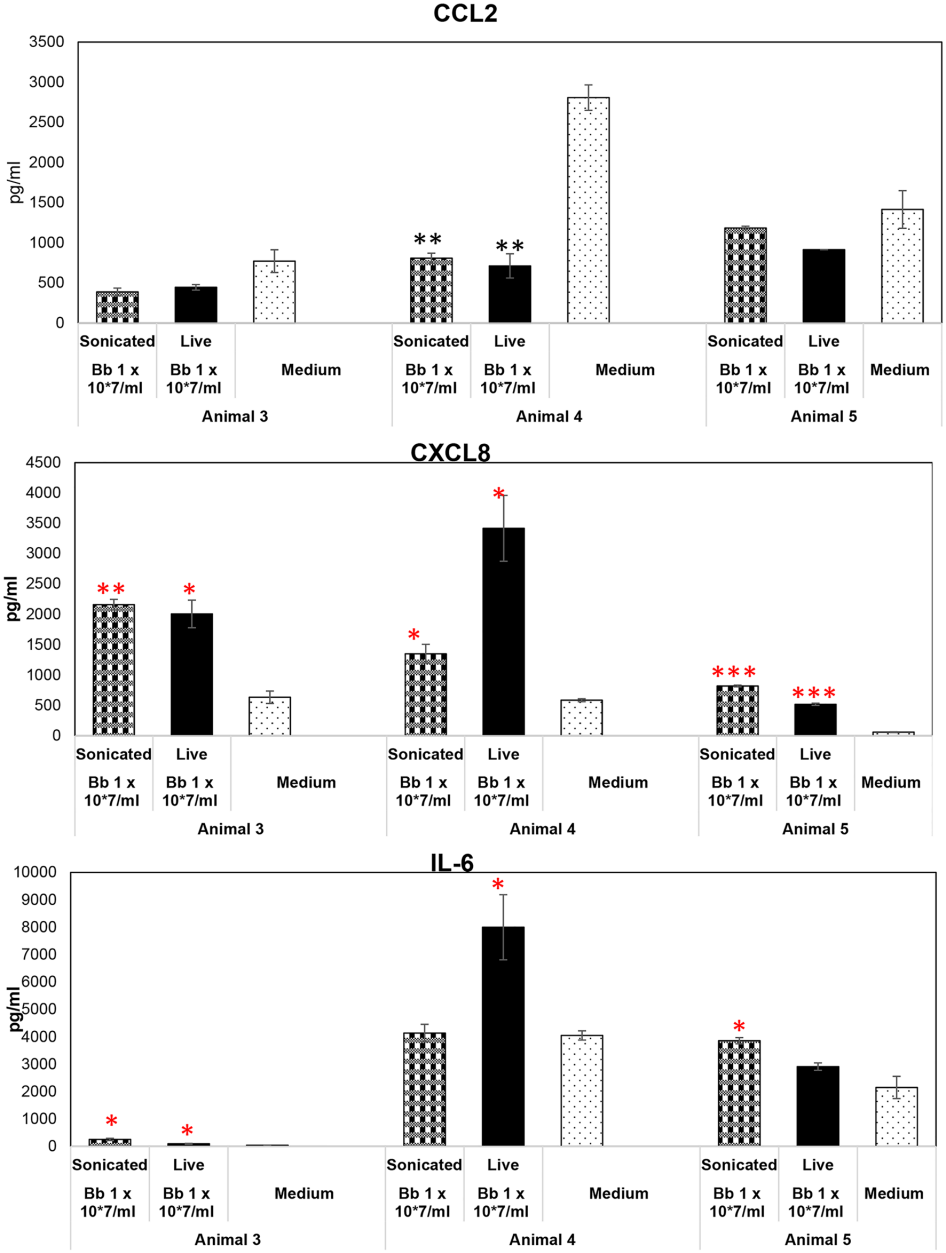
Effect of non-viable *B. burgdorferi* on inflammatory mediator production from the DRG. Rhesus DRG tissues were exposed to live or sonicated *B. burgdorferi* for 4 h. Supernatants were analyzed for CCL2, CXCL-8 and IL-6. Results from tissues obtained from 3 animals is shown. ***p < 0.001; **p < 0.01; *p < 0.05. Significantly increased levels over medium control is denoted by the red asterisks. Black asterisks represent significantly lower levels compared to medium control. Bars represent standard deviation.

Fold change was calculated as levels induced by non-viable *B. burgdorferi* divided by those induced by live bacteria. Values were calculated from levels in Figs. 1 and 2. Numbers greater than 1 indicate higher levels induced by non-viable bacteria, while those less than one indicates higher levels induced by live bacteria. Values that are bold and underlined indicate statistically significant increase in non-viable Borrelia induced levels, while those in bold and italics indicates statistically significant lower levels, compared to those induced by live bacteria. Other values indicate no statistically significant difference. Standard deviation is shown with in brackets. *FC* frontal cortex, *DRG* Dorsal root ganglion. *p < 0.05, **p < 0.01, ***p < 0.001.

### Non-viable *B. burgdorferi* induces inflammatory mediators in primary rhesus Dorsal root ganglion (DRG) tissues

We performed similar experiments in DRG as those described for frontal cortex using 3 animal tissues (Animals 3, 4 and 5). Unlike the frontal cortex, CCL2 was not induced by non-viable *B. burgdorferi* in the DRG in any of the 3 tissues tested (Fig. 2). In fact, levels were lower than those seen in medium controls and significantly so in one of the three. CXCL8 levels were, however, significantly higher than medium levels in all 3 tissues, while significantly higher IL-6 (over medium levels) was seen in 2/3 tissues. Unlike the results in frontal cortex, results with live bacteria showed more concurrence with non-viable bacteria in the DRG. Like sonicated *B. burgdorferi*, live bacteria also did not elicit any CCL2 in the DRG indicating this may be due to host effects than treatment. CXCL8, like those induced by non-viable bacteria, was significantly induced in all three DRG tissues by live bacteria. IL-6 levels induced by live bacteria was higher than medium levels in all three tissues also, while significantly so in 2/3. We then performed similar analysis to those done in FC to ascertain any significant differences in levels induced due to the two treatments. As seen in Table 2B, unlike those seen in frontal cortex, the results from DRG were a bit mixed. Only one of the three animals had higher levels of CCL2 induced by non-viable Borrelia compared to the live bacteria induced levels. With respect to CXCL8, non-viable Borrelia induced higher levels compared to live ones in 1/3, while the live bacteria induced a similar effect in 1/3. IL-6 levels, however, were significantly higher in tissue sections exposed to non-viable Borrelia in 2/3 animals while it was higher in those that received live bacteria in 1/3.

Since FC and DRG were sourced from the same animals numbered 3–5, it is also clear that there are significant differences in tissue specific responses to the same pathogen/antigens. Just taking non-viable bacteria for consideration, FC from animals 3, 4 and 5 induced CCL2 in response to Borrelial fragments, but not in the DRG of the same animals. So, the results thus far indicate that inflammatory response to live Borrelia or its fragments depend on the treatment and tissue source, with non-viable *B. burgdorferi* seemingly more potent inducers than the live bacteria in the brain, while they are somewhat similar in the DRG.

### Are there any inflammatory/cytokine signatures that can distinguish live and non-viable *B. burgdorferi*?

Since the inflammatory mediators tested thus far were induced by both live and non-viable bacteria, we next sought to ascertain if there are any specific inflammatory mediators or cytokines that are induced only by one or the other stimulant that could serve as a signature for the type of antigen (live or dead). We generated a custom 23-plex kit based on the chemokines/cytokines seen in the cerebrospinal fluid (CSF) of patients with Lyme neuroborreliosis (LNB)^17^ and similarly analyzed the supernatants from FC and DRG tissues to determine inflammatory signature differences between the two treatments. The results are shown in Table 3. Of the 23 mediators tested, only 7 were detected in the supernatants of FC in response to treatments (Table 3A). Only FGF2 was significantly secreted in ng/ml quantity, followed by IL-1RA. Levels of all the other 5 were low to modest. Like the previous results in the FC with IL-6, CXCL8 and CCL2 (Fig. 1), there were more tissue samples that significantly induced a mediator with non-viable *Borrelia* than with live ones, but not exclusively so. FGF2 was significantly induced in 2/5 tissues by non-viable *B. burgdorferi* compared to 1/5 with live bacteria. Similar results were observed with GCSF (2/5 vs 1/5), IL-1Ra (3/5 vs 2/5) and SCF (2/5 vs 1/5), while IL-1β, IL-18 and MIP1α were significantly induced by both live and non-viable bacteria in all 5 FC tissues. A similar analysis was also performed in the DRG. Eight mediators were detected in the supernatants of the DRG tissues exposed to either live or sonicated bacteria (Table 3B). However, IL-18 was not secreted in the DRG but IL-17A and VEGF-A were. Lower levels of FGF2 and higher levels of GCSF in comparison to FC induced levels was seen, indicating tissue specific differences in the secretion of mediators but not due to treatment differences. Like the results seen in FC, DRG also did not elicit any inflammatory signature induced exclusively by live or non-viable bacteria. FGF2 and IL-17A were significantly induced in 2/3 DRG tissues in response to sonicated bacteria while 3/3 tissues induced significant levels of these mediators in response to live *B. burgdoferi*. 2 of the 3 tissues induced significant VEGF-A with non-viable bacteria vs 1/3 with live organisms. 1/3 tissues induced significant IL-1RA with sonicated bacteria vs none with live bacteria in the DRG. SCF was significantly induced in only one animal tissue with both live and dead *B. burgdorferi*. However, both treatments elicited significant levels of GCSF, IL-1β, and MIP1α in all 3 tissues, overlapping with the FC results.

**Table 3:** Other significantly induced chemokines/cytokines in the FC and DRG by live and non-viable *B. burgdorferi*.

3A: Frontal cortex tissues										
FC	Animal 1		Animal 2		Animal 3		Animal 4		Animal 5	
	Live	NV	Live	NV	Live	NV	Live	NV	Live	NV
FGF2	3441.9 (± 64.8)	***2596.72*** (± 96.63)	***4603.59*** (± 190.93)	7851.2 (± 702.54)	4184.45 (± 167.83)	**5705.9** (± 461.10)	2304.24 (± 53.56)	***1837.52*** (± 60.68)	**2197.53** (± 4.67)	**1935.10** (± 28.50)
GCSF	ND	ND	ND	ND	**164.01** (± 10.56)	**117.89** (± 0.00)	24.68 (± 8.13)	33.66 (± 4.57)	ND	**60.73** (± 6.70)
IL-1β	**11.69** (± 0.87)	**4.76** (± 0.62)	**11.13** (± 0.90)	**27.99** (± 0.17)	**21.91** (± 1.82)	**14.18** (± 1.68)	**38.99** (± 1.34)	**22.55** (± 2.27)	**42.93** (± 2.00)	**55.42** (± 2.25)
IL-18	**22.75** (± 1.15)	**14.25** (± 0.98)	**11.69** (± 0.25)	**16.16** (± 0.38)	**16.34** (± 1.32)	**9.61** (± 0.98)	**78.66** (± 1.64)	**91.40** (± 3.27)	**25.20** (± 1.32)	**60.90** (± 1.34)
IL-1RA	**447.1** (± 5.12)	**506.95** (± 9.07)	214.10 (± 50.95)	**853.16** (± 30.98)	***581.95*** (± 67.60)	852.12 (± 29.51)	78.53 (± 41.17)	**120.23** (± 42.37)	**344.09** (± 20.4)	170.68 (± 31.54)
MIP1α	**8.74** (± 0.06)	**7.76** (± 0.23)	**11.00** (± 0.06)	**29.28** (± 0.26)	**18.29** (± 0.61)	**32.24** (± 1.14)	**45.11** (± 1.37)	**36.49** (± 0.90)	**50.46** (± 2.07)	**92.29** (± 6.10)
SCF	6.66 (± 0.06)	5.20 (± 1.01)	8.02 (0.00)	10.51 (± 0.65)	3.97 (± 0.20)	3.64 (± 0.66)	3.26 (± 0.93)	**3.17** (± 0.0)	**3.17** (0.00)	**3.265** (± 0.13)

3B: DRG tissues										
DRG	Animal 3		Animal 4		Animal 5					
	Live	NV	Live	NV	Live	NV				
FGF2	**748.74** (± 9.51)	**913.16** (± 63.45)	**357.88** (± 0.90)	**242.96** (± 7.57)	**597.49** (± 4.82)	***344.91*** (± 4.83)				
GCSF	**606.96** (± 15.43)	**815.59** (± 15.88)	**231.3** (± 2.97)	**190.14** (± 6.33)	**57.15** (0.00)	**60.3** (± 14.85)				
IL-1β	**11.42** (± 0.33)	**2.44** (± 0.08)	**8.875** (± 0.66)	**11.13** (± 0.08)	**3.495** (± 0.25)	**1.31** (± 0.35)				
IL-17A	**18.33** (± 0.34)	2.135 (± 0.76)	**13.72** (± 3.46)	**12.99** (± 3.12)	**11.76** (± 2.78)	**11.27** (± 2.10)				
IL-1RA	ND	ND	111.00 (± 28.91)	**224.14** (± 12.46)	ND	ND				
MIP1α	**30.83** (± 0.89)	**20.91** (± 2.18)	**42.71** (± 0.30)	**47.11** (± 2.20)	**5.06** (± 0.04)	**12.22** (± 0.95)				
SCF	**7.40** (± 0.45)	**6.00** (± 0.51)	1.61 (± 0.50)	1.92 (± 0.31)	1.66 (± 0.32)	0.74 (± 0.21)				
VEGF	**63.34** (± 7.79)	**71.97** (± 6.07)	***47.58*** (± 3.14)	***17.19*** (± 0.83)	6.955 (± 1.35)	**25.81** (± 2.54)				

Numbers indicate pg/mL of each chemokine/cytokine with standard deviation in brackets. Significantly higher levels than medium controls are bold and underlined, while those in bold and italics indicate significantly lower levels than medium levels. Other numbers indicate no significant change compared to medium controls. *FC* Frontal cortex, *DRG* dorsal root ganglion, *ND* not detected by the multiplex kit. *NV* non-viable *B. burgdorferi*.

### Inflammatory mediators are induced predominantly in glial cells (and endothelial cells) in response to non-viable *B. burgdorferi*

**Figure 3:**
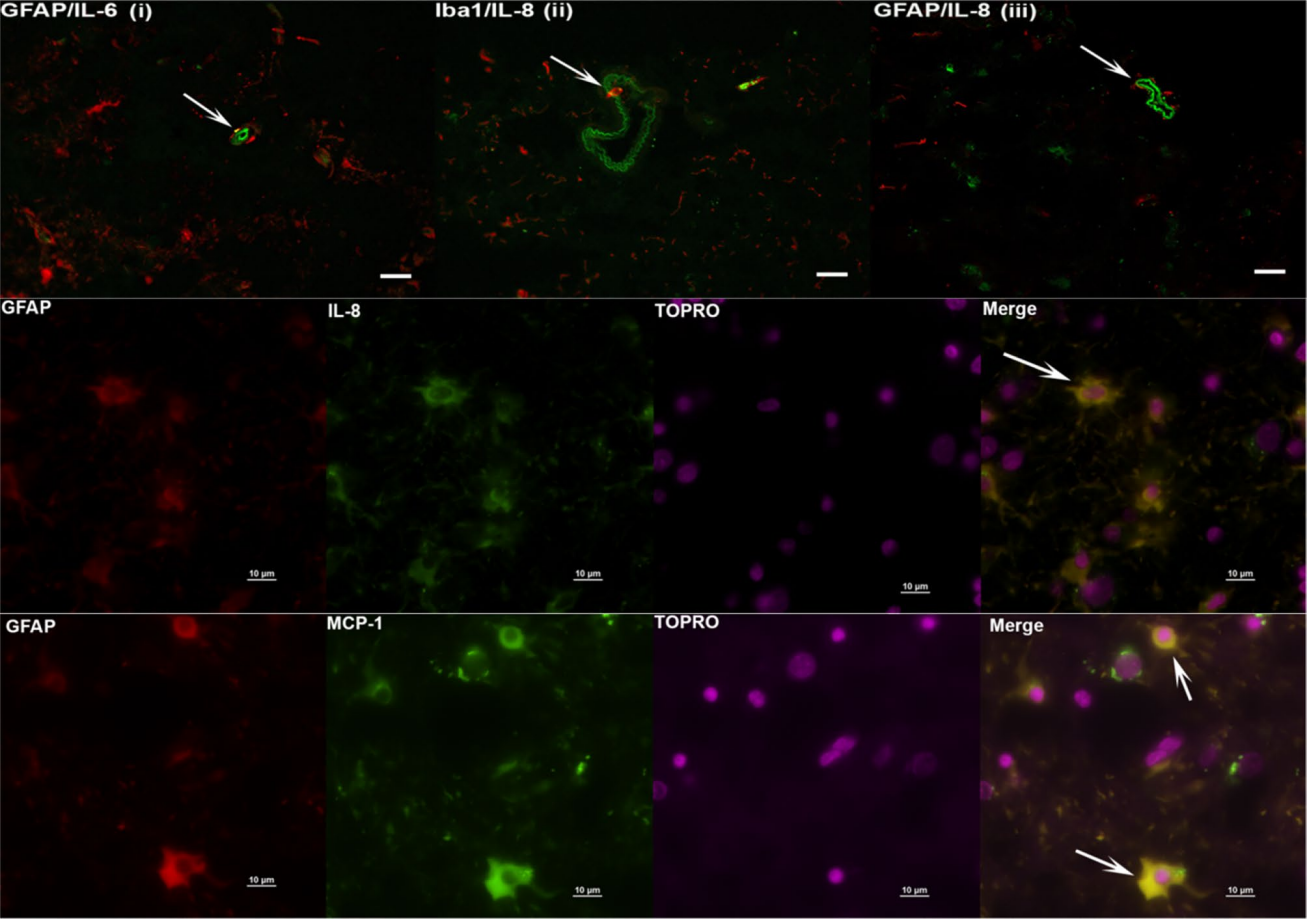
Immunohistochemical staining for inflammatory mediators and brain cells. Tissue sections from Frontal cortex were stained for IL-6, IL-8 and MCP-1, along with cell type specific markers. Top panel (**i**–**iii**) shows IL-6/ IL-8 (green) staining likely within endothelial cells. GFAP or Iba1 are stained in red. Bar represents 25 µm. IL-6 staining in GFAP + astrocytes (**i**), IL-8 staining in Iba1 + microglia (**ii**) are also seen, as yellow colocalization (arrows). In top panel (**iii**) the arrow points to IL-8 staining (green) in likely endothelial cells. Middle Panel shows IL-8 (green) stain in GFAP positive (red) cells co-localizing as yellow. The bottom panel similarly shows MCP-1 staining (green) in astrocytes stained with GFAP (red), resulting in yellow colocalization. Top panel is from Animal 1, middle panel from Animal 2 and bottom panel from Animal 3.

In previous studies, live bacteria were shown to induce specific inflammatory mediators from microglia (IL-1β, CXCL13, Cox 2) and astrocytes (IL-6, IL-8, Cox 2) in the FC^15^ by immunohistochemistry. Likely endothelial cells of the blood vessels were also reported to induce IL-8. In this study we examined the type of cells that responded to non-viable *B. burgdorferi* to induce inflammatory mediator production. We specifically analyzed for IL-6, IL-8 and MCP-1, in the FC and counterstained for oligodendrocytes (MBP), microglia (Iba1), astrocytes (GFAP) and neurons (MAP2). We also analyzed sections from both brefeldin A plus (Bre+) and brefeldin A minus (Bre−) treatments to increase the chances of double staining. The results are shown in Fig. 3.

IL-6 staining was seen in endothelial cells in FC tissues from multiple animals (Fig. 3 top panel, i). Similar IL-6 staining pattern was also seen in sections from medium only tissues. However, there was an approximately twofold increase in the number of endothelial cells staining for IL-6 in tissues exposed to non-viable Borrelia compared to medium controls (not shown). IL-6 staining was also seen in GFAP stained astrocytes surrounding the endothelial cells (yellow colocalization, indicated by white arrow, Fig. 3 top panel, i) and elsewhere in the tissue, along with microglia and oligodendrocytes (not shown). IL-8 staining was seen similarly in endothelial cells of likely lymph or blood vessels (Fig. 3 top panel, ii, iii), and in Iba1 positive cells (arrow, Fig. 3 top panel, ii). (To confirm that these cells were endothelial cells, additional tissue sections were stained for GFAP/Caveolin-1 (1:100, Sigma Aldrich), or GFAP/ von Willebrand factor (1:50, DAKO). Caveolin-1 is highly expressed in endothelial cells, while von Willebrand factor served as an additional marker to identify endothelial cells^18,19^. Based on the staining patterns in similar structures, we concluded that the non-GFAP but IL-6/IL-8 positive, non-Iba1 but IL-8 positive cells in Fig. 3 top panel are likely endothelial cells. IL-8 staining was also seen in GFAP positive cells (Fig. 3, middle panel) while MCP-1 was seen in GFAP positive astrocytes (Fig. 3 bottom panel). We did not see any specific staining in neurons. To summarize, in the FC, IL-6 staining was seen in endothelial cells, astrocytes, microglia and oligodendrocytes, IL-8 in endothelial cells, microglia and astrocytes, MCP-1 in astrocytes.

**Figure 4:**
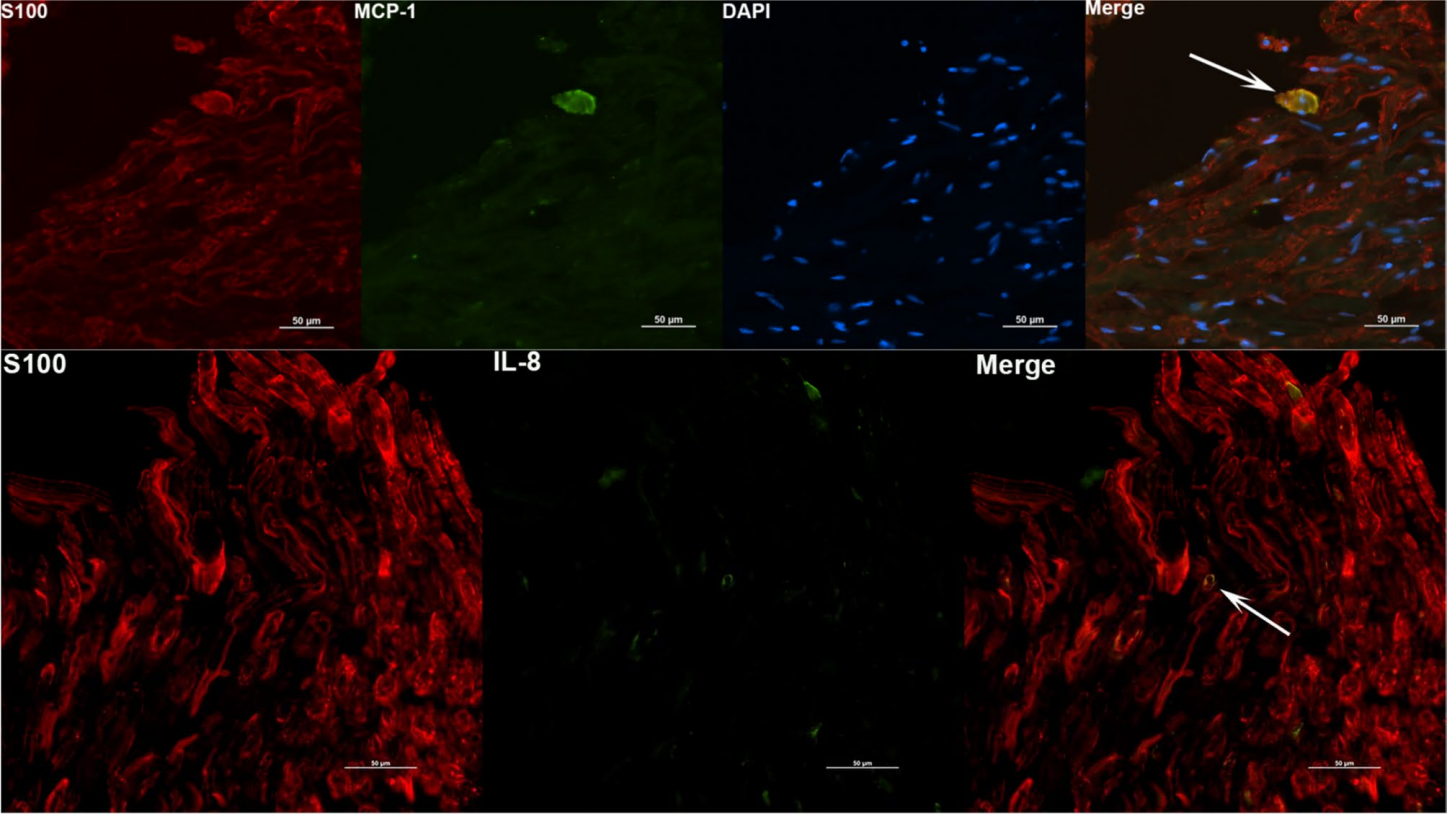
Identity of DRG cells responding to non-viable *B. burgdorferi*. DRG tissues were stained for immune mediators (green) and S100 (red). Positive staining for MCP-1 and IL-8 was seen in S100 positive cells, shown as yellow colocalizations (White arrows). Top panel is from Animal 3 tissue while the bottom panel is from Animal 4 DRG.

Similar analysis was also conducted for DRG tissues, with staining for IL-6, IL-8 and MCP-1 and counterstained with S100 (satellite glial cells, Schwann cells) or NeuN (neurons). The results are shown in Fig. 4. Staining was seen in primarily in the S100 positive cells for MCP-1, IL-8 (Fig. 4, top and bottom panels) and IL-6 (not shown). A few cells that were non S100 also stained for IL-6 (not shown). Staining in NeuN positive cells was not conclusive.

### Non-viable *B. burgdorferi* induces apoptosis in the FC and DRG

**Figure 5:**
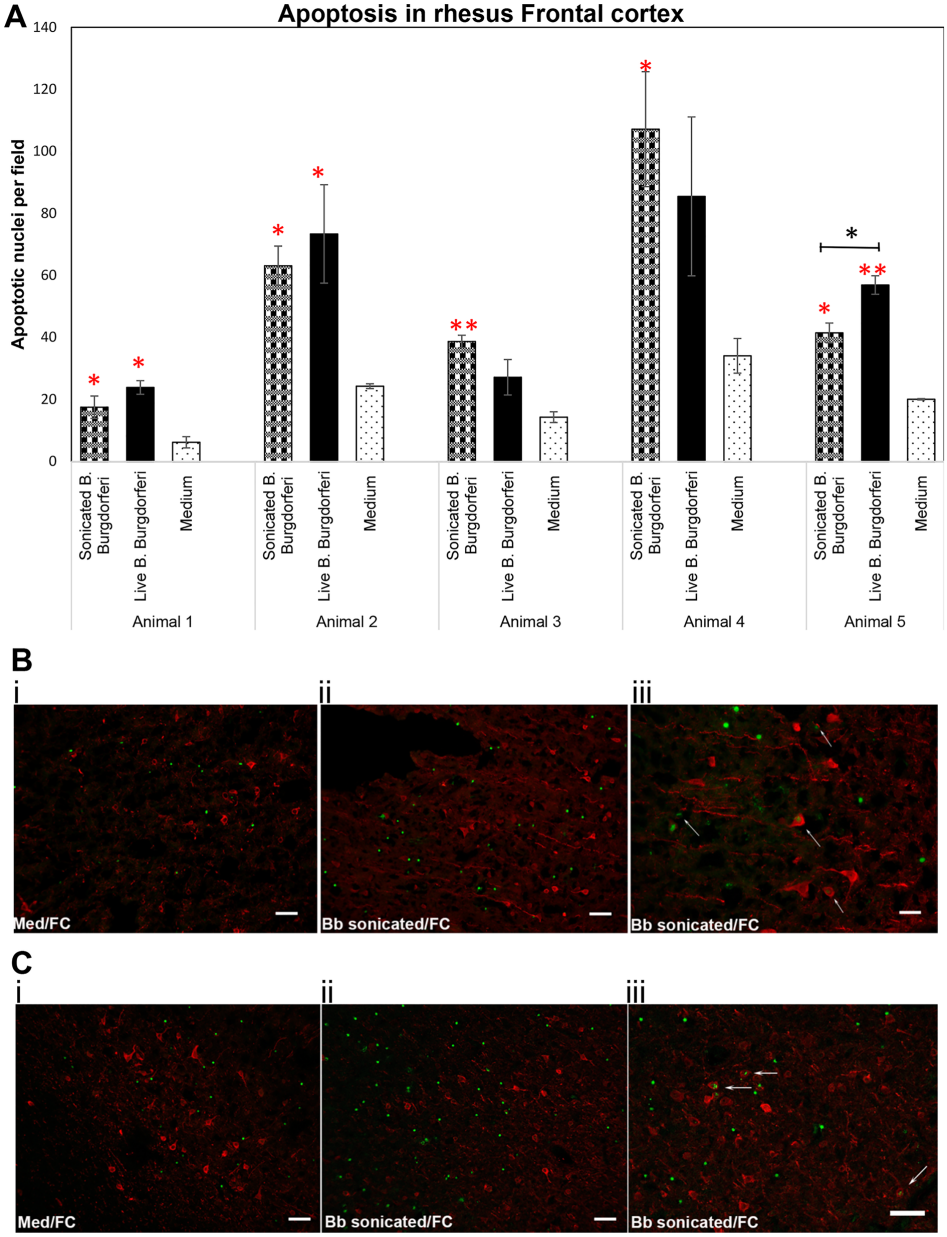
Non-viable *B. burgdorferi* mediates cell death in the rhesus FC. Rhesus frontal cortex tissues were exposed to live or sonicated *B. burgdorferi* for 4 h. TUNEL assays were carried out on cryosections as described in Materials and methods. Results from 5 different animal tissues is shown in (**A**). Bars represent standard deviation. Red asterisks indicate significantly higher levels of apoptosis compared to tissue sections exposed to medium alone. Black asterisk shows significantly higher level of apoptosis in tissues exposed to live *B. burgdorferi* compared to sonicated bacteria in that animal tissue. ***p < 0.001; **p < 0.01; *p < 0.05. Immunofluorescence photographs of TUNEL assays from Animal 1 (**B**) and Animal 2 (**C**). i and ii show the overall difference in apoptotic nuclei (green) between tissue sections exposed to Medium (i) and sonicated bacteria (ii). (iii) shows apoptotic nuclei within MAP2 stained (red) neurons indicated by white arrows. Bar represents 50 µm except for B iii (25 µm).

In previous studies, live *B. burgdorferi* was shown to induce apoptosis in oligodendrocytes and neurons in frontal cortex explants at 4 h^15^. Studies have also shown that this cell death occurs in an inflammatory environment^16,20^. Since non-viable *B. burgdorferi* similarly induced an inflammatory environment in both FC and DRG, we next analyzed their ability to cause cell death by apoptosis. The results are shown in Fig. 5, with live bacteria as a positive control and medium only tissues as a negative control for apoptosis induction. As seen in the Fig. 5A, non-viable *B. burgdorferi* was able to induce apoptosis in all 5 FC tissues, with levels statistically significant over medium controls. Live bacteria as expected, also showed higher levels of apoptotic nuclei in comparison to medium only control tissues, with levels being statistically significant in 3/5 tissues.

**Figure 6:**
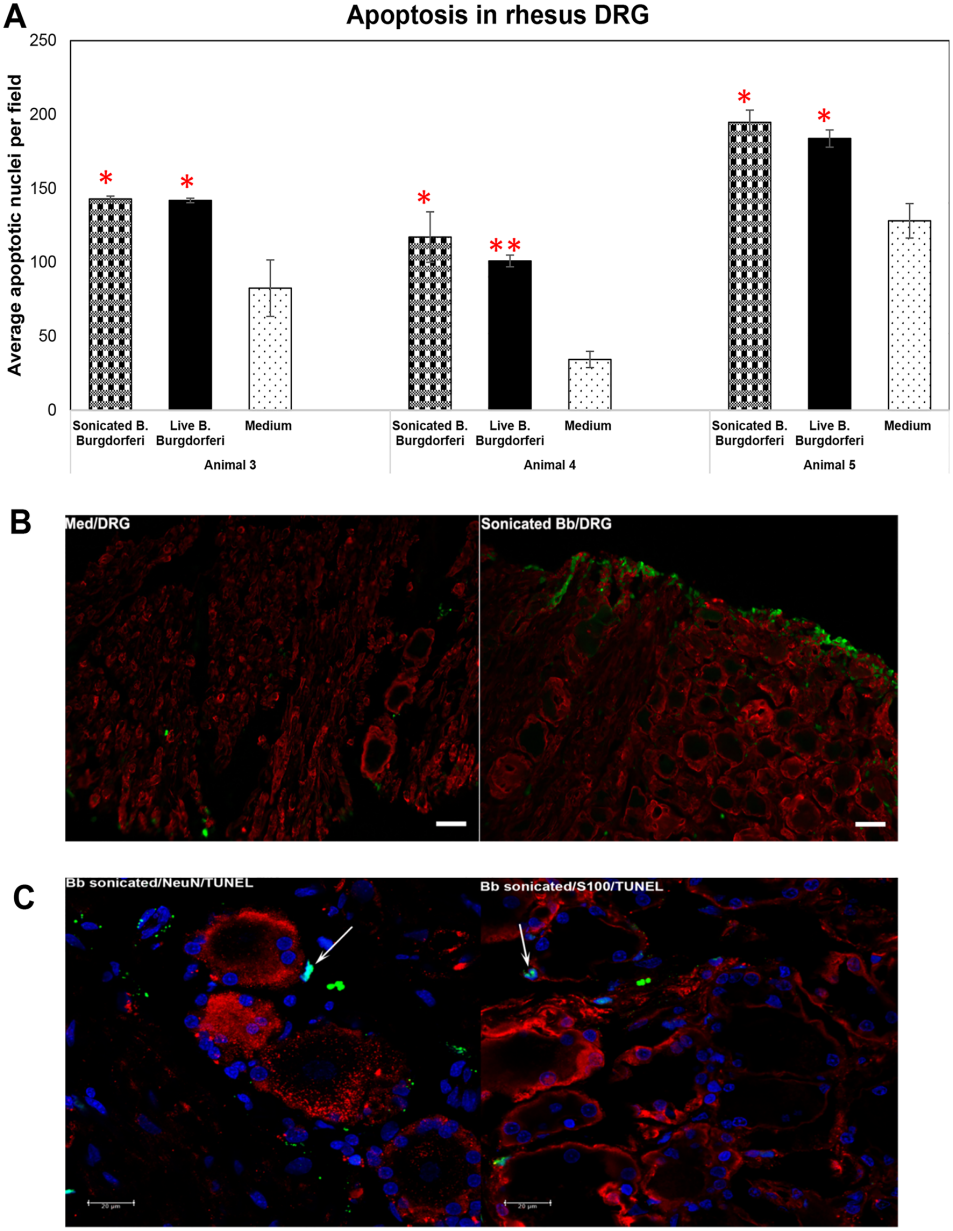
Apoptosis in the rhesus DRG in response to non-viable *B. burgdorferi*. Apoptosis data from three different rhesus DRG tissues in response to live or sonicated *B. burgdorferi* is shown (**A**). Red asterisks indicate statistically significant increases over medium controls. Bar represents standard deviation. (**B**) Immunofluorescence pictures (Animal 4) showing overall increase in apoptosis (green) in tissues exposed to sonicated *B. burgdorferi* in comparison to the tissue sections that received medium only. Bar represents 50 µm. (**C**) confocal images (Animal 5) showing colocalization (cyan, arrows) of TUNEL positive nuclei (green) with DAPI (blue), seen in tissue sections also stained for neurons (NeuN, red) or glial cells (S100, red). Positive apoptotic nuclei are seen only in S100 positive or non-neuronal areas.

Unlike the inflammatory levels, there was not much significant difference in the apoptotic levels induced by non-viable or live *B. burgdorferi* in most tissues, except in tissue from Animal 5 where live bacteria induced apoptosis was significantly higher than that induced by non-viable bacteria (Fig. 5A). We next looked for the type of cells undergoing apoptosis in the FC tissues with non-viable *B. burgdorferi* treatment. Figures 5B and C show apoptotic nuclei predominantly in non-MAP2 cells, (neurons stained for MAP-2 in red), presumably oligodendrocyte glial cells, in response to non-viable *B. burgdorferi*. However, neuronal apoptosis was also seen (Fig. 5Biii,Ciii) in response to sonicated *B. burgdorferi* fragments, albeit at much lower levels in comparison to glial cell apoptosis. Neuronal apoptosis in MAP-2 positive cells was seen in 4/5 tissues in response to non-viable bacteria while live bacteria mediated neuronal apoptosis was seen in 5/5 tissues (not shown). We then performed similar analysis in DRG tissues (Fig. 6). Both live and non-viable *B. burgdorferi* were able to significantly induce apoptosis in all three DRG tissues in comparison to medium only controls (Fig. 6A). No significant difference was observed between apoptosis levels mediated by live or non- viable bacteria in all three tissues. Apoptosis was seen predominantly in S100 positive cells (Fig. 6B,C) in response to non-viable *B. burgdorferi* and in some non NeuN/non S100 cells (not shown). We did not see apoptosis in neuronal cells in any of the three DRG tissues exposed to sonicated *B. burgdorferi* fragments (Fig. 6C), while 1 of the 3 DRG tissue showed neuronal apoptosis in response to live bacteria (not shown).

## Discussion

We show in this study that non-viable *B. burgdorferi* can mediate neuroinflammation and apoptosis (Figs. 1, 2, 5, 6) in primary rhesus FC and DRG tissues ex vivo. The bacterial fragments often elicited higher levels of inflammatory mediators than the live bacteria at the same equivalent concentration. This is likely due to the fact that upon lysis, multiple ligands such as lipoproteins, flagellins, DNA, RNA and others, are released, which can stimulate several TLR receptors^21^, many more than intact bacteria. As bacterial debris likely spreads wider, this could also lead to wider activation of inflammatory signaling pathways resulting in higher levels of inflammatory mediators. This effect was more pronounced in the FC than the DRG and is likely due to the presence of glial cells such as microglia and astrocytes in the CNS. These cells are a significant source of inflammatory mediators, and activation of more of such cells would greatly add to the inflammatory output, as evidenced by the staining of multiple mediators in these cells (Fig. 3). Previous studies have shown that live *B. burgdorferi* can mediate inflammation and apoptosis in FC explants and were used as a positive control^15,20^. However, as seen from Fig. 1, not all FC tissue samples induced significant levels of inflammatory mediators over medium controls in response to live bacteria. At least 2/5 animals did not respond to live bacterial stimulus in mediating CCL2, IL-6 release (Fig. 1) while in the same animal tissues they responded to sonicated bacteria. This indicated that not only do the sonicated bacteria induce higher levels of inflammatory mediators than do live bacteria, they are also capable of being neuroinflammatory even if live bacteria are not. As for the variability of results with live bacteria in comparison to previous studies, rhesus macaques are outbred and not genetically identical. Hence some variability is not unexpected, although they did induce mediators in most tissues. We expected to see increased MCP-1 secretion in the DRG cells. Surprisingly, DRG tissues from all three animals did not elicit any significant levels of MCP-1 in comparison to medium controls, with either live or dead bacteria (Fig. 2). As previous studies have only stained for MCP-1 in DRG tissue and not their levels in the supernatants, it is not clear if this is different from previous results, but if so, it could be again be due to genetic variability^22–24^. Another surprising result was the level of inflammatory mediators induced by the FC vs DRG. FC tissue sections were at least 4 times larger than the DRG sections. Yet when compared within the same animal, the levels induced by DRG sections were higher than those induced in the FC. This is likely because perhaps due to its small size, the multiplicity of infection is higher in the DRG.

Since IL-6, IL-8 (CXCL8) and MCP-1 (CCL2) were the three mediators consistently seen to be secreted in FC tissues, these were the mediators initially tested, and extended to the DRG tissues. We further expanded the repertoire to 23 other chemokines and cytokines based on the data published in CSF of LNB patients^17^. We show that in addition to IL-6, IL-8 and MCP-1, the non-viable bacteria also induced several other chemokines and cytokines, in more tissues than with live bacteria in both the FC and DRG (Table 3). This was undertaken to determine if there were any mediators uniquely induced by either bacterial remnants or by the live ones, and we failed to find any such unique mediator. It is possible that we might find such a mediator through transcriptomics or other omics analysis. Previous studies have demonstrated the presence of IL-10 and VEGF from FC tissues in response to live bacteria^15,20^. However, we did not find them secreted into the supernatants in our study by either treatment, reiterating the variability seen in rhesus macaques just like in humans. Regarding the other mediators that were significantly secreted into the supernatants of FC or DRG tissues (Table 3), IL-1β, IL-18, MIP1α, IL-17A are considered inflammatory proteins^25–27^. Interestingly, IL-17A has been demonstrated to signal through its receptor in rat neurons to promote pain sensitivity^28^. Conflicting roles have been reported for GCSF and FGF2. On the one hand they have both been shown to attenuate neuroinflammation and neuronal apoptosis in rodent models^29–31^. On the other hand, GCSF has been shown to drive inflammation in rheumatoid arthritis and others^32–34^, while FGF2 to induce neuronal apoptosis in primary mouse neuron/glial cultures^35^. VEGF-A, the endothelial growth factor is another molecule demonstrated to have conflicting effects. In rodent models, blocking of VEGF-A has been shown to alleviate painful neuropathies by reducing nociception^36^. On the other hand, a monoclonal VEGF antibody was demonstrated to exacerbate neuropathy and pain in breast cancer patients^37^. SCF (also called Kit ligand) is a mast cell chemotactic factor that mediates mast cell degranulation and histamine release^38^. *B. burgdorferi* has been demonstrated to activate mast cells and release histamine upon coculture with rat or mouse mast cells^39^. Mast cell deficiency in Kit^wsh−/−^ mice enabled faster mobilization of *B. burgdorferi* to the joints, implicating a role in dissemination^40^. IL-1RA on the other hand, is considered to have anti-inflammatory properties as it inhibits IL-1 mediated signaling^41^. While the roles of these individual mediators in *B. burgdorferi* mediated neuroinflammation is yet to be determined, it is likely that both non-viable and live *B. burgdorferi* induce a mix of majority pro-inflammatory factors with a few anti-inflammatory, neuroprotective mediators to counteract the former. However, it is interesting to note that the majority of these mediators such as IL-6, IL-8 (CXCL8), MCP-1(CCL2), IL-1β, IL-18, GCSF, VEGF-A, IL-17A and FGF2 also have one thing in common, in that they have all been demonstrated to be proalgesic, mediating nociception sensitization in various pain conditions^42–46^. It is possible that production of these various mediators has a cumulative effect in inducing pain. Testing the CSF of PTLDS patients with chronic pain or CSF, brain tissue/DRG analysis in relevant in vivo animal models with non-viable *B. burgdorferi* might provide some answers.

The identity of the cells that respond to non-viable *B. burgdorferi* treatment in inducing inflammatory mediators was also examined. As reported with live *B. burgdorferi*^15^, it was predominantly the glial cells that responded to sonicated bacteria. IL-8 and IL-6 staining was also seen in likely endothelial cells in the FC. This has implications for blood brain barrier (BBB) breach. IL-6 can modulate tight junction proteins of endothelial cells in a dose dependent manner, with higher concentrations leading to leakiness of the barrier^47^. Similar effects with increased IL-8 has also been reported^48^. Thus it is possible that in vivo, bacterial fragments can cause breach in BBB and cause increased transport of immune cells, inflammatory mediators or even live bacteria. As a point of fact, in non-human primates, presence of intact bacteria (presumably live, and not killed by antibiotics) have been found in the CNS of antibiotic treated animal^49^. Since the tissues used in this study are short-lived, it is not clear how long the neuroinflammation induced by bacterial remnants can last, and if they are different from live bacteria mediated neuroinflammation in vivo. Interestingly, a previous study by Qin et al. showed that intraperitoneal injection of LPS can cause an increase in CNS TNFα which remained for about 10 months^50^. Given the likely glial cell activation in patients’ brain months after Lyme disease treatment, it is imperative that such studies in vivo are undertaken, to determine whether non-viable *B. burgdorferi* remnants can reach the CNS, and/or cause neuroinflammation. In the DRG, MCP-1 and IL-6 staining has been reported in neurons, satellite glial cells and Schwann cells, and IL-8 in satellite glial cells and Schwann cells in response to live bacteria^22^. In our study, IL-8, IL-6 and MCP-1 staining was seen with S100 positive cells only and not in NeuN stained cells (Fig. 4, and not shown) indicating a glial predominance in inflammatory mediator production. While it is possible that we might have missed neuronal double staining for mediators, it is also possible that neurons do not respond to non-viable *B. burgdorferi*. In vitro primary neuronal cell cultures assays can provide an answer to this possibility. Nevertheless, as DRG are becoming integral to neuropathic pain studies, release of inflammatory mediators in response to sonicated fragments in the DRG has implications for neuropathic pain^14^.

Both glial and neuronal apoptosis were recorded in the FC in response to sonicated bacteria. In the DRG, apoptosis was only seen in S100 positive cells and not in NeuN positive areas in any of the three animal tissues. It is not clear if prolonged incubation would result in neuronal apoptosis, and remains to be investigated either in vitro in primary cultures or in organotypic slice cultures. However, this fact, (neuronal apoptosis in CNS vs lack of in the DRG) along with the more profound inflammatory effect in the CNS indicated that while the non-viable bacteria are pathogenic in both tissues, they are likely more deleterious in the CNS in comparison to the PNS.

In conclusion, we show in this study that non-viable remnants of *B. burgdorferi* are pathogenic to both the CNS and PNS tissues. Presence of chemokines and cytokines in glial cells and endothelial cells in the CNS in response to non-viable fragments has implications for blood–brain barrier breach. Induction of several proalgesic mediators in both the FC and DRG has implications for chronic pain conditions. Persistence of symptoms in some patients post-treatment indicates that in a subset of these patients, *B. burgdorferi* fragments in the nervous system could be a cause. Such antibiotic refractive conditions need novel anti-inflammatory approaches for therapeutics.

## Data Availability

The datasets used and/or analyzed during the current study are available from the corresponding author on reasonable request.

## Abbreviations

BBB: Blood brain barrier
BDNF: Brain derived neurotrophic factor
BLC: B lymphocyte chemoattractant
BSK: Barbour–Stoenner–Kelly
CNS: Central nervous system
CCL2: Chemokine (C–C) motif ligand 2
CCL3: Chemokine (C–C) motif ligand 3
CSF-3: Colony stimulating factor 3
CXCL8: Chemokine (C–X–C) motif ligand 8
CXCL10: (C–X–C) motif chemokine ligand 10
CXCL11: (C–X–C) motif chemokine ligand 11
CXCL13: (C–X–C) motif chemokine ligand 13
DRG: Dorsal root ganglion
ELISA: Enzyme linked immunosorbent assay
EM: Experimental medium
FBS: Fetal bovine serum
FC: Frontal cortex
FGF2: Fibroblast growth factor 2
GCSF: Granulocyte colony stimulating factor
GFAP: Glial fibrillary acidic protein
GMCSF: Granulocyte macrophage colony stimulating factor
IFNγ: Interferon gamma
IL-1β: Interleukin 1-beta
IL-1Rα: Interleukin 1 receptor alpha
IL-4: Interleukin 4
IL-6: Interleukin 6
IL-7: Interleukin 7
IL-10: Interleukin 10
IL-12: Interleukin 12
IL-15: Interleukin 15
IL-23: Interleukin 23
IP-10: Interferon gamma induced protein 10
I-TAC: Interferon-inducible T-cell alpha chemoattractant
LD: Lyme disease
LNB: Lyme neuroborreliosis
MBP: Myelin basic protein
MIP1α: Macrophage inflammatory protein 1 alpha
ND: Not detected
NGFβ: Nerve growth factor bet
NGS: Normal goat serum
NHP: Non-human primate
NV: Non-viable
PBS: Phosphate buffered saline
PET: Positron emission tomography
PNS: Peripheral nervous system
PTLDS: Post-treatment Lyme disease syndrome
SCF: Stem cell factor
TNFα: Tumor necrosis factor alpha
TSPO: Translocator protein
TUNEL: Terminal deoxynucleotidyl transferase dUTP nick-end labeling
VEGF: Vascular endothelial growth factor
